# Bandage-Type Autocatalytic PdCl_2_‑Containing Film as Visual Hydrogen Sensor for Noninvasive Monitoring of Mg-Alloy Biodegradation

**DOI:** 10.1021/acsaom.6c00202

**Published:** 2026-06-11

**Authors:** Juhyeon Park, Michael E. Smith, William R. Heineman, Peng Zhang

**Affiliations:** Department of Chemistry, 2514 University of Cincinnati, Cincinnati, Ohio 45221, United States

**Keywords:** hydrogen gas sensor, magnesium alloy biodegradation, colorimetric detection, autocatalytic hydrogen sensing, noninvasive monitoring, visual gas sensing

## Abstract

We report a bandage-type film for visual hydrogen gas (*H*_2_(*g*)) detection, designed to support noninvasive monitoring of Mg-alloy biodegradation. The sensor consists of PdCl_2_ dispersed in a PEG-40 hydrogenated castor oil matrix, which mitigates drying and enables patch-format use. Upon exposure to *H*_2_(*g*), Pd^2+^ is reduced to Pd^0^, which catalyzes further H_2_ activation, producing an autocatalytic yellow-to-black transition that enables signal quantification by smartphone-based brightness analysis. The film showed no observable response in air and provided a practical linear range of 0–24.6% *H*_2_(*g*) under controlled *H*_2_(*g*) exposures. The device operates in the percent-level *H*_2_(*g*) regime under controlled exposure conditions relevant to Mg-alloy degradation studies. Limits of detection determined at 10, 20, and 30 min were 3.20, 0.98, and 0.49% *H*_2_(*g*), respectively. The formulation has improved storage robustness: the baseline color of the sensing film was maintained for up to 4 weeks in freezer storage, and measurable H_2_ reactivity by the sensing film was retained after up to 4 weeks of storage under ambient, refrigerated, and freezer conditions. Flow-rate testing at 100% *H*_2_(*g*) showed that the response kinetics were independent of flow rate above 10 mL/min. Practical operation of the sensor was demonstrated by detecting H_2_ generated from Mg-alloy that was degraded in phosphate-buffered saline through a chicken-skin overlayer acting as a diffusion-barrier surrogate. This visually readable, autocatalytic bandage-type sensor provides a complementary approach for noninvasive monitoring of Mg-alloy biodegradation.

## Graphical abstract

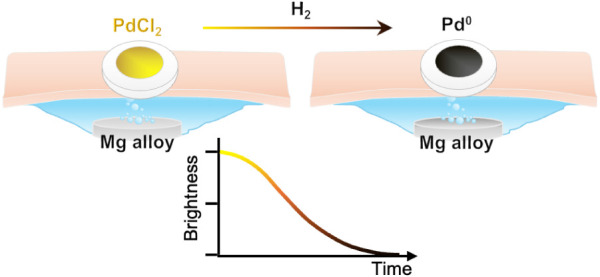

## Introduction

Conventional orthopedic implants such as stainless steel, titanium, and cobalt–chromium alloys have been widely used as permanent fixation devices for applications such as bone fracture fixation.^1–8^ However, their high elastic modulus can induce stress shielding and subsequent bone loss, and long-term implantation may release metal ions/particles that provoke adverse tissue responses.^9–14^ In addition, a secondary surgery is often required for hardware removal, increasing patient burden and healthcare costs. To address these limitations, magnesium (Mg) and Mg alloys have been explored as biodegradable fixation materials that provide more bone-like mechanical properties and gradual resorption in physiological environments.^15–26^ Most importantly, Mg-alloy implants can reduce the need for removal surgery after orthopedic fixation. This consideration is particularly important for pediatric patients with ongoing bone growth. For Mg-based implants, monitoring their biodegradation is essential because the implant must retain sufficient mechanical integrity during bone healing while degrading at a controlled rate thereafter.^13,27^ To evaluate *in vivo* Mg-based implant biodegradation, a range of modalities has been used, including radiography, ultrasonography, microcomputed tomography, synchrotron radiation microcomputed tomography, magnetic resonance imaging (MRI), blood evaluation in combination with inductively coupled mass spectrometry, and histological analyses.^28–30^ These techniques typically provide intermittent rather than real-time information, and several approaches are invasive and require biofluid collection.^28–30^ In addition, these techniques are often costly and resource-intensive and rely on specialized instrumentation and trained operators, making them impractical for frequent monitoring.^28^ These limitations motivate noninvasive, low-cost, and portable approaches for easy monitoring of Mg-implant biodegradation. In aqueous biological fluids, Mg corrosion generates hydrogen gas (*H*_2_(*g*)) (Reaction[Disp-formula eq1]). Thus, the evolution of *H*_2_(*g*) can serve as an indicator of Mg degradation.

R1 $$\mathrm{Mg}\;+\;2H_{2}O\to {\mathrm{Mg}}^{2+}+2{\mathrm{OH}}^{-}+H_{2}$$

While many Mg-based orthopedic devices were first introduced and adopted outside the United States, some have started to receive U.S. FDA authorization/clearance in recent years, further motivating practical tools to monitor their biodegradation in clinical settings.^31,32^ Beyond implant monitoring, sensitive *H*_2_(*g*) detection is important for safety considerations because hydrogen is colorless and odorless and therefore difficult to detect by human senses, necessitating dedicated leak detection strategies.^33^ Hydrogen measurement is also clinically relevant, as hydrogen breath testing has been explored for gastrointestinal disorders, including carbohydrate malabsorption and small intestinal bacterial overgrowth (SIBO).^34^

Our group has been developing visual *H*_2_(*g*) sensing films as an instrument-free (i.e., no dedicated analytical instrumentation required) approach for point-of-need hydrogen monitoring.^35,36^ In our prior dye-based platform, *H*_2_(*g*) was detected through the reduction of resazurin to resorufin, producing a blue-to-pink color transition in the presence of Au–Pd or Pd nanoparticles serving as catalysts.^35–38^ These reagents were incorporated into water-rich polymer matrices (e.g., agarose/alginate hydrogels or PDADMAC) to provide optically transparent, hydrated environments that facilitate uniform color development and visual signal.^35,36^ Other dye/catalyst-based visual H_2_ indicators have also been advanced by other groups, including resazurin/Au–Pd nanoparticle supraparticle systems designed for bare-eye H_2_ indication and recording.^39^ Separately, *in vivo* studies have demonstrated that H_2_ generated from Mg-based implant biodegradation can be measured transdermally using electrochemical and visual sensing approaches, supporting the physiological relevance of through-skin H_2_ monitoring.^40,41^ Together, these studies motivate complementary optical approaches for simple, visual, and noninvasive H_2_ monitoring without specialized instrumentation. However, in our previous water-rich dye-based film format, practical patch-style deployment was limited by photothermal instability of the representative dyes (e.g., resazurin, bromothymol blue, methyl red) and by drying of water-type matrices at ambient conditions over time, which can markedly reduce or eliminate *H*_2_(*g*) reactivity. In addition, the incorporated nanoparticle catalysts in these dye-based systems may accelerate baseline color change during storage, complicating handling and long-term stability. To improve robustness while maintaining a visual response, we have therefore transitioned to PdCl_2_-based colorimetry (Reaction[Disp-formula eq2]), in which PdCl_2_ (yellow) is reduced by *H*_2_(*g*) to Pd^0^ (black).

R2 $${\mathrm{Pd}}^{2+}\left(\mathrm{yellow}\right)+H_{2}\to {\mathrm{Pd}}^{0}\left(\mathrm{black}\right)+2H^{+}$$

The Pd^0^ formed in situ can promote H_2_ activation and accelerate further PdCl_2_ reduction, enabling an autocatalytic colorimetric response without the need for an external nanoparticle catalyst.^37,38^ PdCl_2_ was selected as the hydrogen-responsive component because palladium-based systems are well established for H_2_ activation,^42–44^ and Pd/PdCl_2_-containing materials have been used in optical or gasochromic H_2_ sensing systems.^45,46^ In our formulation, PdCl_2_ also provided the most rapid visually measurable response under room-temperature conditions. To mitigate drying and enable coformulation of an aqueous PdCl_2_ phase within an oil-rich film, we replaced the water-based polymer matrix (e.g., PDADMAC/hydrogel used in our prior dye-based sensors) with PEG-40 hydrogenated castor oil (polyethylene-glycol 40 hydrogenated castor oil, PEG-40 HCO).^47^ PEG-40 HCO is an amphiphilic, nonionic solubilizer/emulsifier widely used to disperse hydrophilic and hydrophobic components within a single formulation and to maintain a paste-like, nondrying matrix at ambient conditions.^48^ Moreover, it is derived from hydrogenated fatty-acyl chains and therefore largely saturated, making direct chemical consumption of *H*_2_(*g*) by the matrix unlikely under our ambient, catalyst-free sensing conditions. Accordingly, PEG-40 HCO primarily functions as a nonionic carrier/solubilizer (and mild plasticizer) that mitigates drying-induced cracking, stabilizes the local microenvironment for dissolved Pd^2+^, and maintains efficient *H*_2_(*g*) transport within the film to facilitate Pd^2+^ reduction and the associated optical response.^49^ Using this formulation, we have developed a laminated, bandage-type visual *H*_2_(*g*) sensing film designed to monitor hydrogen evolution from Mg-alloy biodegradation. The device integrates PdCl_2_-based colorimetric chemistry within a drying-resistant, oil-rich matrix to support patch-format visual monitoring of *H*_2_(*g*) permeation in Mg-alloy degradation studies.

## Experimental Section

### Materials, Reagents, and Instrumentation

Palladium­(II) chloride (PdCl_2_; Acros), PEG-40 hydrogenated castor oil (PEG-40 HCO; Mystic Moments), and polydimethylsiloxane (PDMS; SYLGARD 184, Dow) were used to prepare the bandage-type sensing film. Phosphate-buffered saline (PBS, 10×, pH 7.4; Fisher Scientific) and deionized water (≥18 MΩ·cm) were used to prepare aqueous solutions. Ultrahigh-purity N_2_ and H_2_ gases (Wright Brothers) were used with calibrated gas proportioning rotameters (Omega Engineering) for preparing defined *H*_2_(*g*) conditions (Supporting Information, Figure S1 and Table S1). Hydrogen concentrations were measured using an electrochemical H_2_ microsensor (Unisense) with a Unisense Microsensor Multimeter. A complete list of reagents, common laboratory supplies/consumables, and part numbers (e.g., tapes, tubing, fittings, Petri dishes, and balloons) used in this work, is provided in the Supporting Information (Experimental Section S1).

### Fabrication of the Bandage-Type Visual *H*_2_(*g*) Sensing Film

To improve suitability for on-skin use, a compact bandage-type sensor was fabricated (Figure 1). The device (overall diameter 20.0 mm) was assembled as a laminated stack comprising (1) a gas-permeable skin-contact tape (3M 9834), (2) an adhesive spacer/bonding layer (3M 1577), (3) a polydimethylsiloxane (PDMS) holder containing the sensing mixture, and (4) a white backing layer (3M 9907W) to maximize optical contrast (Figure 1A). The PDMS holder and sensing-cavity geometry were fabricated using an aluminum plate mold as shown in Figure S2. The sensing matrix was confined to a circular region (10.0 mm diameter) within the PDMS holder. The sensing mixture (70 μL) was dispensed into the holder and immobilized by brief freezing prior to lamination to preserve uniform thickness and minimize lateral spreading (Figure 1B). The filled volume slightly exceeded the nominal cavity volume because the flexible PDMS holder can deform, creating additional interfacial space during assembly.

**Figure 1:**
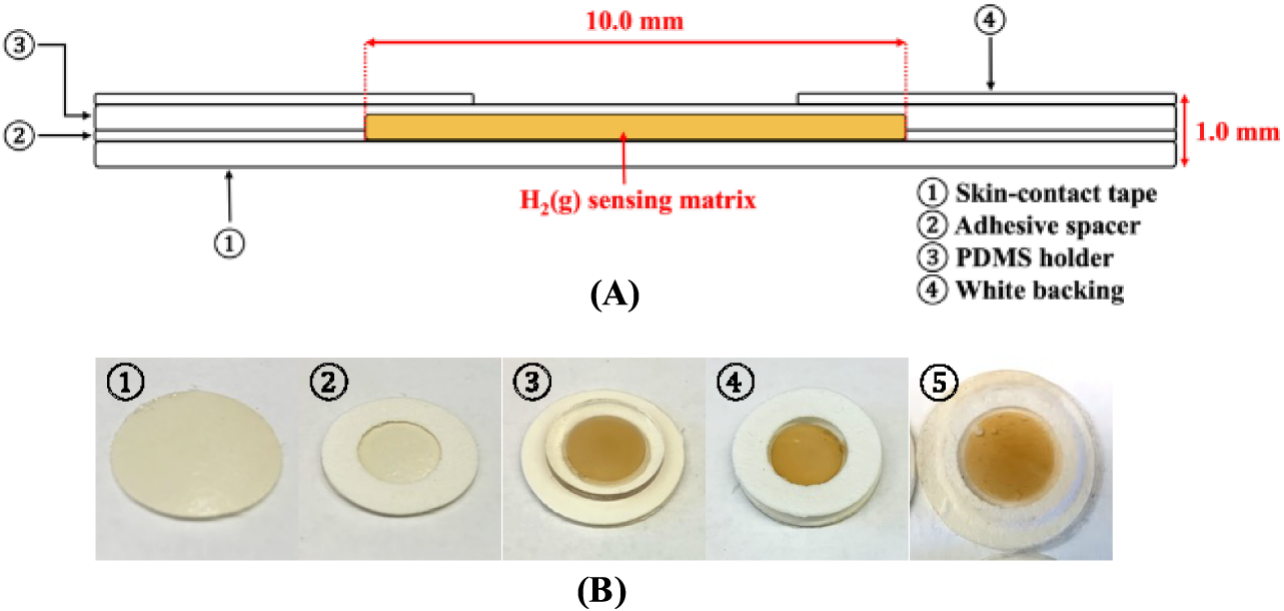
Fabrication and structure of the bandage-type *H*_2_(*g*) sensing film. (A) Cross-sectional schematic of the laminated device (not to scale). The *H*_2_(*g*) sensing matrix is confined within a PDMS holder and sandwiched between a gas-permeable skin-contact layer and a white backing layer to enhance optical contrast. The sensing region diameter is 10.0 mm, and the holder thickness is ∼1.0 mm. The main components are (1) gas-permeable, skin-contact tape, (2) adhesive spacer, (3) PDMS holder, and (4) white backing tape. (B) Representative assembly sequence images showing (1) PDMS holder preparation, (2) bonding of the adhesive spacer, (3) loading of the sensing mixture, (4) sealing with the white backing, and (5) the final assembled device. Overall device diameter: 20.0 mm.

### Preparation of the PdCl_2_/PEG-40 HCO Sensing Mixture

PEG-40 HCO (52.5 μL; 0.0525 g) was dispensed into a PDMS thin-layer holder, followed by addition of 17.5 μL of 26.8 mM PdCl_2_ in 1× PBS (prepared in deionized water). The resulting PdCl_2_ concentration in the mixture was 6.7 mM. This composition corresponds to approximately 25 vol % 1× PBS dispersed within the PEG-40 HCO matrix, providing an aqueous environment for PdCl_2_ solubilization while maintaining a nondrying bulk phase. The holder was briefly warmed on a 40 °C hot plate for 2 min to reduce viscosity and facilitate homogeneous dispersion of the aqueous PdCl_2_ phase within the PEG-40 HCO matrix. The mixture was then gently mixed using a pipet tip to avoid bubble formation. No visible color change was observed during this preparation step. The filled holders were stored in a freezer prior to lamination to temporarily immobilize the sensing mixture, minimizing lateral spreading and preserving uniform thickness during device assembly.

### RGB-Based Image Analysis for Optical Response Quantification

The color change of the visual *H*_2_(*g*) sensing film was quantified by RGB-based image analysis. ImageJ software was used to extract region-of-interest (ROI)-averaged RGB values from time-lapse images. A square ROI (170 × 170 pixels) was placed at an identical fixed location within the sensing area for all time points and samples. Brightness (B) was defined as the mean of the ROI-averaged R, G, and B channels. To minimize the influence of ambient illumination, the sensing ROI brightness was normalized by the brightness of a white background reference region within the same frame (eq 3), yielding the normalized brightness ratio (B_t_/B_o_), where B_o_ is the initial brightness at t = 0. Images were acquired under fixed camera-to-sample distance and constant lighting. They were analyzed directly from the original image files. Detailed imaging conditions are provided in the Supporting Information. All optical response measurements were performed in triplicate unless otherwise specified (n = 3). Apparent optical response rates were obtained from the slope of B_t_/B_o_ versus time over the specified time window.

1 $${\mathrm{Brightness of H}}_{2}(g)\;\mathrm{sensing film}\;\left(B\right)=\frac{{\mathrm{RGB average of H}}_{2}(g)\;\mathrm{sensing film}}{\mathrm{RGB average of white background}}$$

Detailed fabrication protocols, gas-mixing configurations, balloon-based tests, calibration procedures, statistical analysis methods, and supplementary validation experiments are described in Supporting Information.

## Results and Discussion

### Balloon-Based Test of *H*_2_(*g*) Reactivity and RGB-Based Quantification

The laminated bandage-type architecture (Figure 1) confines the *H*_2_(*g*)-responsive mixture to a defined optical window and uses a white backing to maximize contrast, enabling robust quantification of the normalized brightness response (B_t_/B_o_) from time-lapse images. The reactivity of the bandage-type *H*_2_(*g*) sensing film was first validated using a *H*_2_(*g*)-filled balloon, coupled with RGB-based image analysis for response quantification. Since *H*_2_(*g*) permeates through the balloon latex very slowly, the *H*_2_(*g*)-filled balloon serves as a simple, reproducible *H*_2_(*g*) source. Upon contact with the *H*_2_(*g*)-filled balloon, the PdCl_2_/PEG-40 HCO sensing layer exhibited a clear yellow-to-black transition (Figure 2), consistent with Pd^2+^ reduction to Pd^0^ as described in Reaction[Disp-formula eq2].

**Figure 2:**
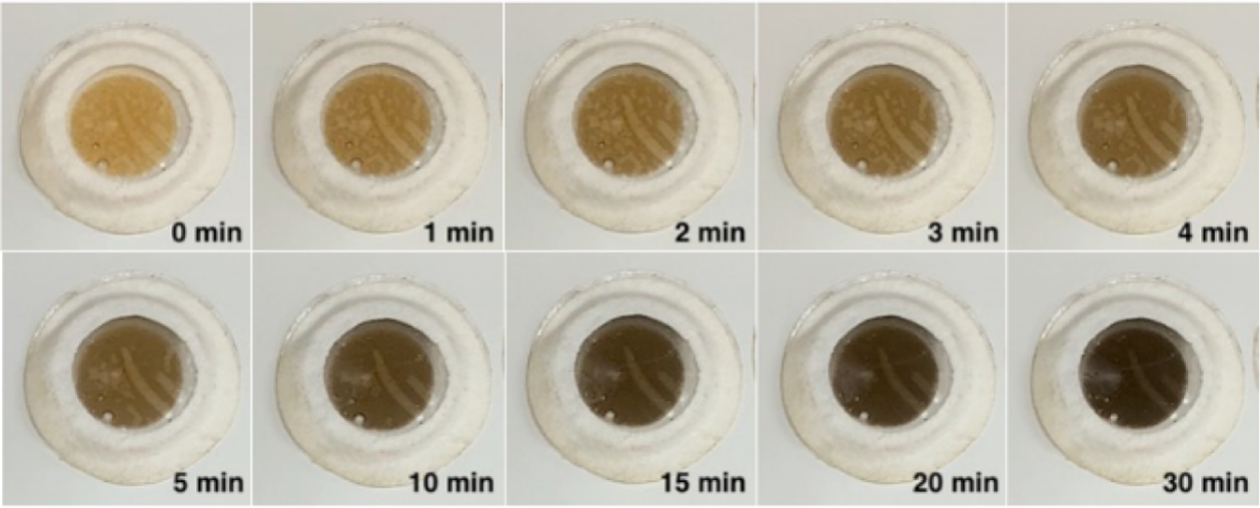
Representative optical response of the bandage-type *H*_2_(*g*) sensing film on an *H*_2_(*g*)-filled balloon. Time-lapse images show a progressive transition from yellow to black in the PdCl_2_/PEG-40 HCO sensing mixture upon exposure to *H*_2_(*g*), consistent with the reduction of Pd^2+^ to Pd^0^. Images are arranged in chronological order (left to right): top row, 0–4 min; bottom row, 5–30 min.

Time-lapse images as shown in Figure 2 were processed to obtain the normalized brightness ratio (B_t_/B_o_) as defined in eq 3, with results shown in Figure 3. Under 100% *H*_2_(*g*)-filled balloon exposure, B_t_/B_0_ decreases rapidly at the early stage and then transitions to a slower regime, consistent with a transport-limited progression as the PdCl_2_-containing matrix is reduced. For standardized quantitative comparisons, we extracted an apparent early stage response slope from the near-linear 0–6 min window by linear regression of B_t_/B_o_ versus time. This fixed 0–6 min window provides a practical and reproducible response metric for quantifying the apparent reaction rate. This fixed-window approach minimizes later-time (after 6 min) saturation effects when comparing data under different conditions. Together, the normalized B_t_/B_o_ traces and early stage slope analysis establish a practical method for quantifying optical response by RGB analysis.

**Figure 3:**
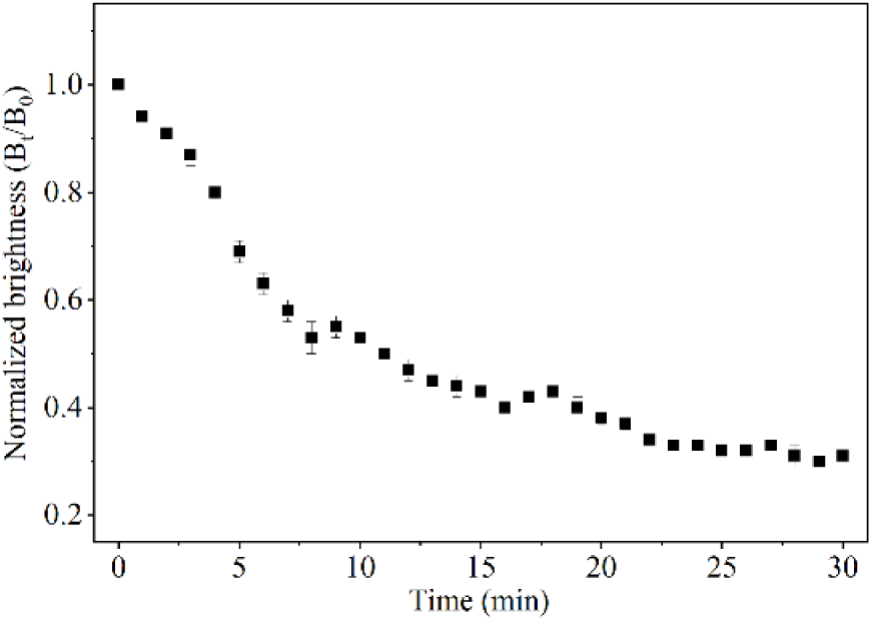
RGB-based quantification of the optical response of the visual *H*_2_(*g*) sensing film. Time-dependent normalized brightness ratio (B_t_/B_o_) extracted from ROI-averaged RGB values upon exposure to *H*_2_(*g*). The response shows a rapid decrease at the early stage (0–6 min), followed by a slower regime, consistent with reaction- and transport-limited behavior during PdCl_2_ reduction. Error bars represent the standard deviation (*n* = 3).

### Evidence for an Autocatalytic PdCl_2_ Reduction Pathway

To evaluate whether the optical response kinetics are consistent with Pd^0^ -mediated acceleration, we compared apparent early stage response rates extracted from two consecutive time windows (0–3 and 3–6 min). As summarized in Table S2, the magnitude of the normalized brightness decay rate is higher in the 3–6 min window, indicating faster PdCl_2_ reduction as the reaction progresses. This rate acceleration is kinetically consistent with an autocatalytic behavior, in which the Pd^0^ species formed in situ promote further H_2_ activation and PdCl_2_ reduction (Reaction[Disp-formula eq4]).^37,38^


R3

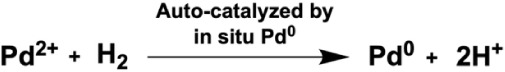




Reaction R3. Proposed autocatalytic pathway for PdCl_2_ reduction mediated by Pd^0^ formed in situ.

### Air-Filled Balloon Control Experiment

As an air-exposure control, the visual *H*_2_(*g*) sensing mixture was exposed to an air-filled balloon, and B_t_/B_o_ was tracked for 30 min under the same imaging and analysis conditions used for *H*_2_(*g*) testing. No visible darkening was observed during air exposure (Figure 4A), and the corresponding B_t_/B_o_ trace remained essentially constant over the 30 min window (Figure 4B). Although this air-filled balloon control does not represent a comprehensive specificity evaluation, these results indicate no observable response of the visual *H*_2_(*g*) sensing mixture to air under the tested conditions.

**Figure 4:**
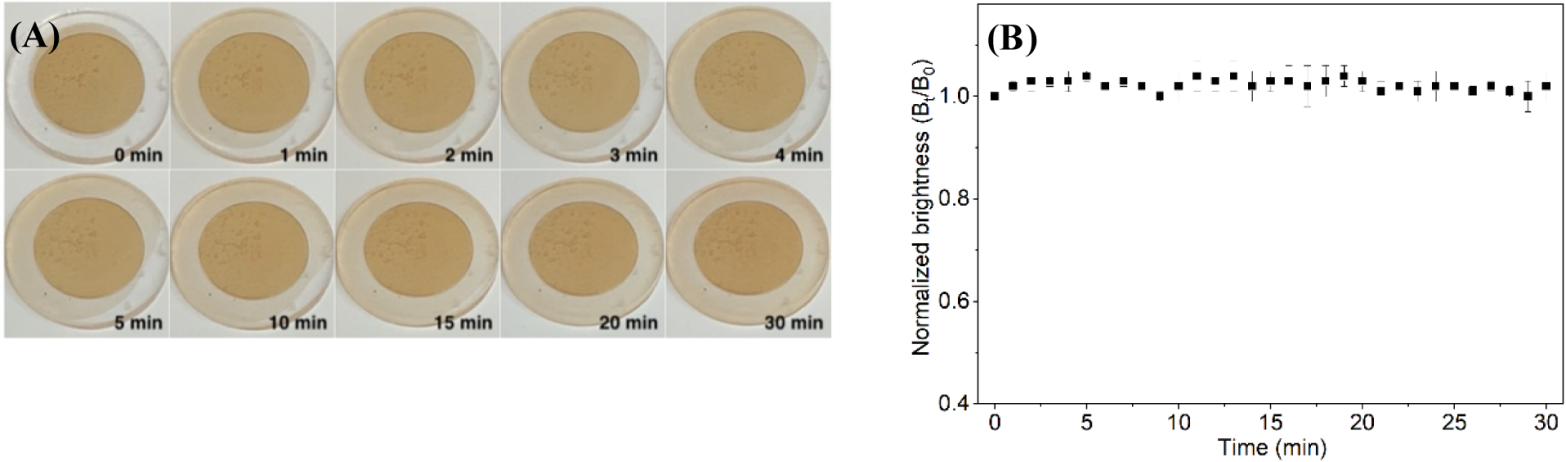
Air-filled balloon control experiment. (A) Time-lapse photographs of the bandage-type sensing film on an air-filled balloon, showing no discernible color change over 30 min under the same contact geometry used for *H*_2_(*g*) testing. Images are arranged chronologically from left to right (top row: 0–4 min; bottom row: 5–30 min). (B) Corresponding normalized brightness (B_t_/B_o_) trace extracted from ROI-averaged RGB values, remaining near unity throughout air exposure. Error bars represent the standard deviation of triplicate measurements (*n* = 3).

### Effect of Storage Temperature on Sensor Stability

To assess storage-temperature-dependent stability of the visual *H*_2_(*g*) sensing mixture, samples were stored at room temperature (22.0 °C), in a refrigerator (2.4 °C), or in a freezer (−17.3 °C) and evaluated at weeks 1–4 relative to the fresh condition (n = 3 per time point). Representative images (Figure 5) show that baseline darkening was more apparent at higher storage temperatures, becoming visually noticeable approximately 3 weeks after storing at room temperature and in the refrigerator, whereas samples stored in the freezer retained their initial appearance over the full 4-week period.

**Figure 5:**
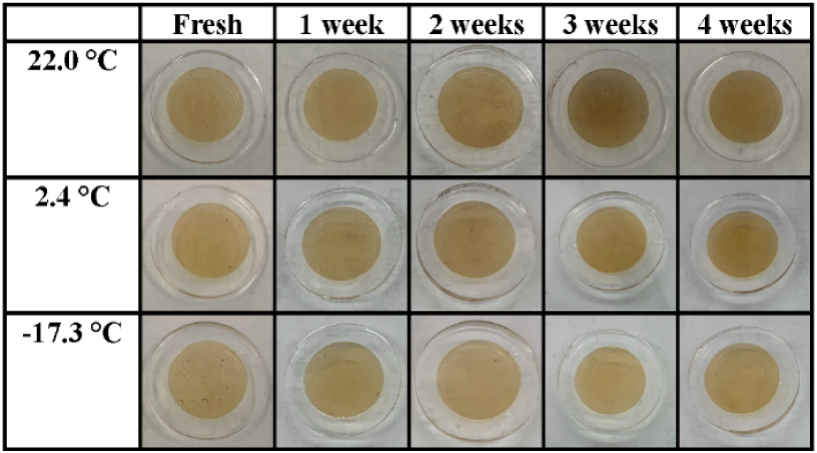
Effect of storage temperature on the baseline color (brightness) of the visual *H*_2_(*g*) sensing mixture. Representative photographs are shown for freshly prepared samples (Fresh) and those stored at room temperature (22.0 °C), in a refrigerator (2.4 °C), or in a freezer (−17.3 °C) after 1, 2, 3, and 4 weeks.

Quantitative analysis of the normalized optical response (Fresh = 1.000) supports these observations (Table S3). When the sensors were stored at room temperature, one-way ANOVA indicated a significant effect of storage time (p = 0.00197). Tukey’s multiple-comparisons test showed that the normalized brightness (B_t_/B_o_) was significantly lower than the fresh condition at week 3 (p = 0.00189) and week 4 (p = 0.01671), whereas week 1 (p = 0.72482) and week 2 (p = 0.09498) were not significantly different from Fresh. When the sensors were stored in the refrigerator, one-way ANOVA also indicated a significant effect of storage time (p = 0.02203). In this group, a significant decrease relative to Fresh was observed only at week 4 (p = 0.02970), whereas week 1 (p = 0.59382), week 2 (p = 0.96084), and week 3 (p = 0.08248) were not significantly different from Fresh. In contrast, sensors stored in the freezer showed no significant effect of storage time by one-way ANOVA (p = 0.33302), and no statistically significant differences from Fresh were observed across all weeks tested (week 1, p = 0.64739; week 2, p = 0.99799; week 3, p = 0.43096; week 4, p = 0.53584), consistent with the minimal visual change shown in Figure 5.

### Retained *H*_2_(*g*) Reactivity After Storing under the Tested Storage Conditions

Although storage temperature might affect baseline appearance (Figure 5), the sensing mixtures retained measurable *H*_2_(*g*) reactivity after storage, as evaluated using a balloon model (Figure 6). To evaluate storage-dependent *H*_2_(*g*) reactivity beyond baseline appearance, sensors stored under different conditions were tested on a *H*_2_(*g*)-filled balloon and analyzed by the aforementioned B_t_/B_o_ analysis method. Representative images confirm that sensors stored for 3 weeks at room temperature retained a measurable optical response upon *H*_2_(*g*) exposure (Figure 6A), and the corresponding B_t_/B_o_ traces show clear time-dependent darkening (Figure 6B).

**Figure 6:**
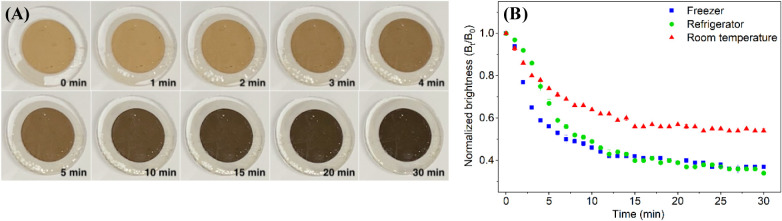
*H*_2_(*g*) reactivity after storing under the tested storage conditions. (A) Representative photographs showing the time-dependent darkening of the visual *H*_2_(*g*) sensing mixture upon exposure to an *H*_2_(*g*)-filled balloon after 3 weeks of storage at room temperature. Images are arranged chronologically from left to right (top row: 0–4 min; bottom row: 5–30 min). (B) Normalized brightness (B_t_/B_o_) versus time traces for sensors stored for 3 weeks under room temperature (22.0 °C), in a refrigerator (2.4 °C), and in a freezer (−17.3 °C) conditions. Experiments were performed in triplicate (*n* = 3).

Apparent early stage rates extracted using the standardized window (as described in Figure 3) are summarized in Table S4 at each storage condition for Weeks 1–4. Overall, freezer storage best preserved sensor reactivity across the full 4-week period, whereas room-temperature and refrigerator storage showed greater variability and more pronounced performance loss at later-time points. Practically, these results suggest that cold storage (especially freezing) is advantageous for maintaining both baseline appearance and response performance; nevertheless, measurable *H*_2_(*g*) reactivity is retained even after storing at room temperature for at least 3 weeks under the tested storage conditions.

In a separate test, two sensing-mixture samples stored at room temperature in vacuum-sealed Mylar bags largely retained similar normalized brightness after approximately 60 days (Figure S7). This suggests that sealed packaging helps mitigate baseline color drift during ambient-temperature storage. For practical handling, these results support storing the sensing mixture or assembled films in vacuum-sealed packaging when possible and visually checking the baseline color before use; pronounced baseline darkening or drying would indicate that the film should not be used for H_2_ exposure assessment.

### Detection of Mg Alloy Degradation-Derived *H*_2_(*g*) through a Chicken-Skin Barrier

We next tested the sensor performance using a biologically derived diffusion barrier by placing chicken leg skin over an MgGd_5_ alloy specimen immersed in 1× PBS and attaching the sensing film to the outer (air-exposed) skin surface. A skin-only control (no MgGd_5_) was measured in parallel. The test with the MgGd_5_ shows a clear time-dependent darkening of the sensing film, whereas the control remains essentially unchanged (Figure 7A), indicating that the observed optical response originates from MgGd_5_ degradation-derived hydrogen rather than nonspecific effects such as humidity or the skin substrate.

**Figure 7:**
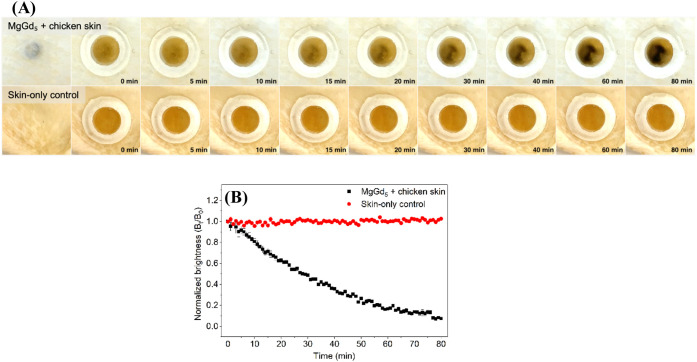
Detection of MgGd_5_ degradation-derived H_2_ through a chicken-skin barrier using the visual *H*_2_(*g*) sensing film. (A) Time-lapse photographs of the sensing film attached to the outer (air-exposed) surface of a piece of chicken skin covering a MgGd_5_ specimen immersed in 1 × PBS; a control (skin-only, no MgGd_5_) is shown for comparison. The leftmost column shows the underlying skin for the MgGd_5_-containing setup and the skin-only control. Images are arranged chronologically from left to right (0–80 min), with the top row corresponding to the MgGd_5_+chicken skin and the bottom row to the skin-only control. (B) Corresponding time-dependent normalized brightness ratio (B_t_/B_o_) extracted from ROI-averaged smartphone images for the MgGd_5_+chicken skin and skin-only control. Error bars represent the standard deviation of triplicate measurements (*n* = 3).

Image analysis based on photographs taken by a smartphone shows a time-dependent decrease in B_t_/B_o_ for the sensing film placed on the skin surface with MgGd_5_ underneath, while the control remains near unity (Figure 7B). This result demonstrates that the sensing film can visually report *H*_2_(*g*) evolution associated with Mg-alloy degradation even when a barrier such as skin is present, similar to hydrogen evolution being a characteristic product of Mg-alloy corrosion in physiological environments.^56,57^ Notably, measurements from the electrochemical sensor indicated higher local *H*_2_(*g*) levels (converted using the polynomial calibration in Figure S4A) in the chicken-skin configuration than in the balloon configuration (Figure 7 and Figures S5–S6). Yet the apparent optical kinetics on chicken skin were slower than on the balloon surface when comparing within the same brightness window (B_t_/B_0_ from 1.0 to 0.6), which is consistent with transport-limited delivery of *H*_2_(*g*) to the film surface. Specifically, the chicken-skin experiment yielded y = −0.019x + 0.99, corresponding to an estimated time of ∼20.5 min to reach B_t_/B_0_ = 0.6, while the balloon experiment yielded y = −0.061x + 1.02, corresponding to ∼6.9 min to reach B_t_/B_0_ = 0.6 (Figures 3 and 7B). This indicates an approximately 3-fold slower apparent response for the chicken-skin configuration. In this geometry, *H*_2_(*g*) generated in 1× PBS must permeate across the liquid–gas interface and traverse the intervening skin layer before reaching the sensing film, introducing additional resistance that can reduce effective trans-skin flux even if local accumulation occurs beneath the barrier. This transport-limited behavior is consistent with prior descriptions of gas accumulation and cavity formation associated with Mg corrosion environments^58^ and with the diffusion-layer sensitivity of Clark-type amperometric measurements.^59,60^ Thus, the electrochemical signal and optical kinetics reflect different physical observables (local accumulation versus surface-averaged flux), and their divergence under barrier conditions is physically reasonable rather than contradictory. Taken together, Figure 7 demonstrates that the sensing film remains functional in the presence of a biologically derived diffusion barrier. Note that chicken skin was used as a practical, biologically derived surrogate to introduce transport resistance and heterogeneity, not intended to quantitatively reproduce human transdermal flux and microvascular clearance in perfused tissue.^61^ Rather, this configuration serves as a qualitative stress test confirming that the film remains responsive under diffusion-limited conditions relevant to Mg-alloy corrosion environments.

### Balloon-Based LOD Measurement under Controlled %*H*_2_(*g*) Atmospheres

To evaluate the practical limit of detection (LOD) of the visual *H*_2_(*g*) sensing film under controlled and reproducible gas-phase conditions, balloon-based tests were used to generate different %*H*_2_(*g*) atmospheres. Defined *H*_2_(*g*) atmospheres (0, 11.7, 24.6, 47.6, and 100% *H*_2_(*g*)) were prepared in a balloon by adjusting *H*_2_(*g*) and *N*_2_(*g*) flow-meter scale settings while maintaining the same total gas volume for each composition (Table S6). This constant-volume approach minimizes pressure- and delivery-related effects, enabling an unbiased evaluation of the optical response and the LOD. The sensing film was placed directly on the balloon surface, and the time-dependent normalized brightness ratio (B_t_/B_0_) was quantified from images (Figure S8); the response magnitude was defined as ΔB = 1 – (B_t_/B_0_). LOD was calculated using the IUPAC criterion, LOD = 3σ­(blank)/m, where σ­(blank) is the standard deviation of ΔB measured at 0% *H*_2_(*g*) (n = 3), and m is the slope of the calibration line. Because LOD depends on both the blank variability and the calibration slope, we report LOD values using a fixed analysis workflow (ROI size, normalization, and time window) and restrict the calibration range to where linearity is statistically supported (Table S5) to avoid artificially optimistic detection limits. Linearity was assessed across potential calibration ranges (0–24.6%, 0–47.6%, and 0–100% *H*_2_(*g*)) using the root-mean-square error (RMSE) and R^2^ (Table S5). Across all response windows, the lower-range calibration (0–24.6%) provided the most reliable linear behavior (higher R^2^ and lower RMSE compared to the wider ranges that include higher *H*_2_(*g*) levels). Using the 0–24.6% range, the estimated LOD values were 3.20% (0–10 min), 0.98% (0–20 min), and 0.49% *H*_2_(*g*) (0–30 min) (Table S5), indicating that longer response windows improve sensitivity. Based on the 0–20 and 0–30 min windows, a practical and conservative LOD of ∼1% *H*_2_(*g*) (range: 0.49–0.98%, depending on the response window) is supported. For comparison, these %*H*_2_(*g*)-based LOD values can be expressed on an H_2_-equivalent dissolved-concentration scale by applying the independent microsensor calibration that relates %*H*_2_(*g*) to [*H*_2_(*g*)]­eq (μM) (Figure S4), corresponding to approximately 4.00–4.27 μM (1% ≈ 4.05 μM). We recognize that these LOD values should be interpreted as controlled gas-phase exposure limits for the balloon-based measurement configuration, while *in vivo* detectability will also depend on implant geometry, degradation rate, tissue transport, and local H_2_ clearance.

### Flow-Rate Effect on the Optical Response Kinetics under 100% *H*_2_(*g*)

To assess whether the time-dependent optical response of the *H*_2_(*g*) sensing film is influenced by gas-delivery conditions, we varied the 100% *H*_2_(*g*) flow rate delivered to a Franz-cell test fixture from 10 to 89 mL min^–1^ (10, 32, 61, and 89 mL min^–1^; 10 mL min^–1^ was the minimum accessible setting in our current gas-delivery configuration) and tracked the normalized brightness ratio (B_t_/B_o_) over 0–15 min (Figure S9; Table S7). Analysis of covariance (ANCOVA) showed the significant effect of time (*p* < 0.0001), but no significant effect of flow rate (p = 0.218) or time × flow-rate interaction (p = 0.246), suggesting that the time-dependent optical response profiles were statistically indistinguishable across the tested flow rates. These results indicate that, within the tested range of flow rates, the apparent optical response kinetics were not detectably altered by *H*_2_(*g*) flow rate. Importantly, the accompanying electrochemical measurements obtained under the same setup show that the H_2_ level associated with the different flow settings spans a broad range (Table S9; ∼154 μM at 10 mL min^–1^ up to ∼561 μM at 89 mL min^–1^), yet the brightness–time trajectories remain statistically indistinguishable (Figure S9). Together, these results suggest that once the delivered H_2_ reaches the flow rate of ≥∼10 mL min^–1^ in our setup (Table S9), the observed color-change rate is governed primarily by the intrinsic film chemistry (i.e., a flow-rate-independent response) rather than being limited by bulk gas-delivery rate. This control experiment is valuable because dynamic gas-sensor measurements can, in general, be sensitive to experimental hydrodynamic factors (e.g., residence time, convection–diffusion boundary layers), so similar responses observed across the tested flow-rate range help reduce the possibility that the reported kinetics/LOD arise primarily from a bulk gas-delivery artifact.^62^ Because flow rates below 10 mL min^–1^ were not accessible with the present configuration, different behavior remains possible in that lower-flow regime and is not ruled out by this data set.

## Conclusion

In this work, we report the development of a bandage-type visual *H*_2_(*g*) sensor that integrates PdCl_2_-based colorimetry within a drying-resistant PEG-40 hydrogenated castor oil matrix for visual monitoring of hydrogen evolution from biodegradable Mg alloys. The sensing chemistry leverages Pd^0^-mediated autocatalytic reduction of Pd^2+^ by *H*_2_(*g*), producing a distinct yellow-to-black transition without requiring external catalysts, while the oil-based matrix mitigates drying and enhances usability in skin-interfacing formats. The film showed no observable response under air exposure over the tested time window and good storage stability: the initial color was maintained after up to 4 weeks of freezer storage, and measurable *H*_2_(*g*) reactivity was retained after up to 4 weeks of storage under room-temperature, refrigerator, and freezer conditions. Patch-format functionality was tested with a biologically derived diffusion barrier by detecting *H*_2_(*g*) generated from Mg-alloy degradation in PBS through a chicken-skin overlayer, demonstrating that the sensing film retains functionality under diffusion-limited barrier conditions. Based on image analysis on photographs taken by a smartphone, the film achieved a controlled exposure LOD of 0.49–0.98% H_2_(*g*) for 20–30 min response windows, corresponding to approximately 4.00–4.27 μM on a H_2_-equivalent dissolved-concentration scale derived from independent electrochemical microsensor measurements. Finally, flow-rate testing at 100% H_2_(*g*) showed statistically indistinguishable response kinetics between 10 and 89 mL min^–1^ by ANCOVA, suggesting that above the minimum flow rate (10 mL min^–1^) in our setup, the observed color-change rate is governed primarily by intrinsic film chemistry rather than bulk gas-delivery rate. Overall, this PdCl_2_-based sensing film provides a visually readable, autocatalytic H_2_ sensing platform with good storage stability and functionality in a diffusion-barrier configuration. These findings establish a practical basis for noninvasive monitoring of Mg-alloy biodegradation and suggest broader opportunities for point-of-need H_2_ detection in settings where percent-level H_2_(*g*) exposure can be assessed on site.^63,64^

## Supplementary Material



## Notes

Experimental Section S1: materials, reagents, and instrumentation; Experimental Section S2: fabrication of the PDMS thin-layer holder; Experimental Section S3: gas-mixing setup and *H*_2_(*g*) concentration measurement; Experimental Section S4: stability study design and statistical analysis; Experimental Section S5: stability of the sensing mixture (*H*_2_(*g*) reactivity) under different storage conditions; Experimental Section S6: balloon-based test of H_2_(g) reactivity; Experimental Section S7: detection of *H*_2_(*g*) from MgGd_5_ alloy degradation through a chicken-skin barrier; Experimental Section S8: preparation of balloons with various %*H*_2_(*g*) for testing; Experimental Section S9: balloon-surface optical measurement and image analysis for LOD; Experimental Section S10: effect of *H*_2_(*g*) flow rate on optical response kinetics; Figure S1: gas-flow apparatus used in *H*_2_(*g*) calibration and balloon experiments; Table S1: manufacturer-provided flow-rate data for N_2_ and H_2_ (GPR-N_2_ and GPR-H_2_) and corresponding theoretical H_2_ concentrations; Figure S2: aluminum plate mold for preparing the PDMS thin-layer holder; Figure S3: manufacturer-provided flow-rate calibration curves for N_2_ and H_2_ (GPR-N_2_ and GPR-H_2_); Figure S4: calibration curve relating amperometric H_2_ sensor potential (mV) to H_2_-equivalent dissolved concentration on an H_2_(g)-filled balloon; Figure S5: representative amperometric H_2_ sensor potential (mV) traces on an H_2_(g)-filled balloon; Figure S6: representative amperometric H_2_ sensor potential (mV) measured on chicken skin over MgGd_5_ in 1× PBS; Table S2: linear analysis of optical response of the visual *H*_2_(*g*) sensing film; Table S3: storage-temperature-dependent stability of the normalized optical response; Table S4: *H*_2_(*g*) reactivity after storage; Figure S7: test of H_2_-sensing mixtures stored in vacuum-sealed Mylar bags at room temperature; Figure S8: time-dependent normalized brightness (B_t_/B_0_) under 0–100% H_2_(g) for LOD analysis; Table S5: linearity metrics (RMSE, R²) and IUPAC LOD values from ΔB at different response windows and %H_2_(g) ranges; Table S6: flow settings for preparing different H_2_(g)/N_2_(g) mixtures balloon atmospheres (LOD experiments); Figure S9: time-course of B_t_/B_0_ under 100% H_2_(g) at different flow rates (ANCOVA); Table S7: conversion of rotameter scale readings to 100% H_2_(g) flow rates used in the different flowrate experiment; Table S8: balloon-based calibration for converting %H_2_(g) to H_2_-equivalent concentration, [*H*_2_(*g*)]_eq_ (μM); Table S9: measured [H_2_]­eq (μM) under different *H*_2_(*g*) flow rates in the Franz cell setup (PDF)
