# Supplementary material for: Bandage-Type Autocatalytic PdCl2‑Containing Film as Visual Hydrogen Sensor for Noninvasive Monitoring of Mg-Alloy Biodegradation
Source: ACS Appl Opt Mater. 2026 Jun 11;4(6):1851–60. doi: 10.1021/acsaom.6c00202 (PMC13316995; doi:10.1021/acsaom.6c00202)
Supplement: Supplementary file 1 [file ot6c00202_si_001.pdf]

Supporting Information

Bandage-Type Autocatalytic $PdCl_2$-containing Film as Visual Hydrogen Sensor for Noninvasive Monitoring of Mg-Alloy Biodegradation

Juhyeon Park, Michael E. Smith, William R. Heineman*, Peng Zhang*

Department of Chemistry, University of Cincinnati, Cincinnati, OH 45221, USA

*Professor William R. Heineman

Phone: 513-556-9210

Email: heinemwr@ucmail.uc.edu

*Professor Peng Zhang

Phone: 513-556-9222

Email: zhangph@ucmail.uc.edu

Contents

Experimental Section S1. Materials, reagents, and instrumentation (Expanded list) S-4,5

Experimental Section S2. Fabrication of the PDMS thin-layer holder. S-5

Experimental Section S3. Gas-mixing setup and H_2(g) concentration measurement. S-5

Experimental Section S4. Stability study design and statistical analysis. S-6

Experimental Section S5. Stability of the sensing mixture (H_2(g) reactivity) under different storage conditions. S-6

Experimental Section S6. Balloon-based test of H_2(g) reactivity. S-6

Experimental Section S7. Detection of H_2(g) from $MgGd_5$ alloy degradation through a chicken-skin barrier. S-6

Experimental Section S8. Preparation of balloons with various %H_2(g) for testing. S-7

Experimental Section S9. Balloon-surface optical measurement and image analysis for LOD. S-7

Experimental Section S10. Effect of H_2(g) flow rate on optical response kinetics. S-7

Figure S1. Gas-flow apparatus used in H_2(g) calibration and balloon experiments. S-8

Table S1. Manufacturer-provided flow-rate data for N_2 and H_2 (GPR-N_2 and GPR-H_2) and corresponding theoretical H_2 concentrations S-8

Figure S2. Aluminum plate mold for preparing the PDMS thin-layer holder. S-9

Figure S3. Manufacturer-provided flow-rate calibration curves for N_2 and H_2 (GPR-N_2 and GPR-H_2) S-9

Figure S4. Calibration curve relating amperometric H_2 sensor potential (mV) to H_2-equivalent dissolved concentration on an H_2(g)-filled balloon S-10

Figure S5. Representative amperometric H_2 sensor potential (mV) traces on an H_2(g)-filled balloon. S-10

Figure S6. Representative amperometric H_2 sensor potential (mV) measured on chicken skin over $MgGd_5$ in 1× PBS. S-11

Table S2. Linear analysis of optical response of the visual H_2(g) sensing film S-11

Table S3. Storage-temperature-dependent stability of the normalized optical response. S-11

Table S4. H_2(g) Reactivity after storage. S-12

Figure S7. Test of H_2-sensing mixtures stored in vacuum-sealed Mylar bags at room temperature. .. S-12

Figure S8. Time-dependent normalized brightness (B_t/B_0) under 0–100% H_2(g) for LOD analysis. .. S-13

Table S5. Linearity metrics (RMSE, R^2) and IUPAC LOD values from ΔB at different response windows and %H_2(g) ranges. S-13

Table S6. Flow settings for preparing different H_2(g)/N_2(g) mixtures balloon atmospheres (LOD experiments). S-14

Figure S9. Time-course of B_t/B_0 under 100% H_2(g) at different flow rates (ANCOVA). S-14

Table S7. Conversion of rotameter scale readings to 100% H_2(g) flow rates used in the different flow-rate experiment S-15

Table S8. Balloon-based calibration for converting %H_2(g) to H_2-equivalent concentration, $[H_2(g)]_{eq}$ (µM). S-15

Table S9. Measured $[H_2]_{eq}$ (µM) under different H_2(g) flow rates in the Franz cell setup S-16

References. S-16

Experimental Section S1. Materials, Reagents, and Instrumentation (Expanded List)

Chemicals and materials were used as received. Aqueous solutions were prepared using deionized (DI) water (≥18 MΩ·cm).

S1.1. Chemicals and polymers

Phosphate-buffered saline (PBS, 10×, pH 7.4) and a 3-way valve made of polytetrafluoroethylene (PTFE) were purchased from Fisher Scientific (NJ). Palladium(II) chloride, 59% Pd ($PdCl_2$, AC195200010) was purchased from Acros. PEG-40 hydrogenated castor oil (500 g) was purchased from Mystic Moments Store. A polydimethylsiloxane (PDMS) silicone elastomer kit (SYLGARD 184) was purchased from Dow Corning (MI).

S1.2. Gases and gas-delivery components

N_2 and H_2 gases of ultra-high purity grades were purchased from Wright Brothers (OH). A gas proportioning rotameter (GMR2-010343) with two flow tubes (FL-2GP-61C-61C) was obtained from Omega Engineering (CT). A single flow tube rotameter (FP 1/8-038-G-6) was purchased from Lab-Crest, Fisher & Porter (PA). Stainless steel tubing (1/8 in o.d.) was purchased from Supelco (PA). A stainless-steel reducing union (1/4 in to 1/8 in, part #SS-400-6-2) was obtained from Swagelok (OH). White PTFE tape (part #6802K14) and double-sided, double-lined silicone adhesive tape (part #7213A27) were acquired from McMaster-Carr (OH). Nitrile rubber O-rings (1/4 in i.d., 1/2 in o.d., part #9452K183) were acquired from McMaster-Carr (OH). Tygon tubing (1/4 in i.d., 3/8 in o.d., part #6516T21) and PTFE tubing (3/16 in i.d., 1/4 in o.d., part #5239K12) were acquired from McMaster-Carr (OH).

S1.3. Electrochemical H_2(g) measurement

Amperometric H_2(g) sensing probes (H2-ST-1/4-304204; blunt tip) and a multimeter were purchased from Unisense (Denmark).

S1.4. Device-fabrication materials and consumables

An aluminum plate (3/16 in thick, part #3003-H14) was obtained from Metals Depot (KY) and used to prepare the aluminum plate mold. Petri dishes (60 mm, polystyrene) were purchased from Nest (China). 1577 Adhesive Polyester Medical Tape, Medical Tape 9834 (single-sided polyurethane, 78# carrier, configurable), and Medical Tape 9907W (single-sided elastic nonwoven fabric, 55# liner, configurable) were acquired from 3M (MN). White balloons (10 in) were purchased from Rubfac Store. Corning Falcon bacteriological Petri dishes (CLS351008; 35 mm × 10 mm) were purchased from MilliporeSigma (OH).

S1.5. Mg alloy specimen and biologic barrier materials

$MgGd_5$ alloy (disc shape; 5 mm diameter and 2 mm thickness) was provided by the University of Pittsburgh, Swanson School of Engineering and School of Dental Medicine. Fresh, uncooked chicken drumsticks were purchased at Kroger (OH).

S1.6. Common laboratory equipment and software

Standard laboratory items used in this work included micropipettes and pipette tips, a hole punch (15 mm diameter), and a 6-well tissue-culture plate used to maintain mold alignment during PDMS curing. A hot plate was used for mixture homogenization. Time-lapse videos were acquired using the rear camera of an iPhone XR positioned approximately 10.0 cm above the sensing film. Videos were recorded at 1080p resolution and 30 frames/sec using the default iPhone Camera app settings, with the camera-to-sample distance and imaging geometry kept constant throughout each experiment. The sensing-film region was tapped on the screen before recording to focus on the sensing area. Images were acquired in the same laboratory hood using the hood's built-in white light as the illumination source, the intensity of which was not measured. No additional manual exposure, white-balance, or color-correction settings were applied. Still-frames used for RGB analysis were extracted from the original video files without post-acquisition color correction. ImageJ (version 1.54g) was used for RGB/region-of-interest analysis, and OriginPro (version 2026) was used for statistical analyses. A Franz cell-based test fixture (flattened Franz cell) was used for measurements under flowing gas conditions (see Figure S1).

Experimental Section S2. Fabrication of the PDMS thin-layer holder. A PDMS thin-layer holder was prepared to contain the H_2(g) sensing mixture. An aluminum plate mold with a central protrusion was machined for this purpose (Figure S2A); the protrusion defined the sensing-cavity diameter ($d_{protrusion}$ = 10 mm) and depth ($h_{protrusion}$ = 0.6 mm), and the mold base diameter was 33 mm (d_{base} = 33 mm). The mold was sized to fit in a 6-well plate to maintain alignment during curing (Figure S2B). To prepare one holder, SYLGARD™ 184 base (1.35 g) and curing agent (0.15 g) were mixed thoroughly (10:1, w/w) and cast on the mold. This mold-defined cavity was used to standardize the sensing-matrix thickness across devices prior to lamination into the bandage-type sensor (Figure 1). The total mass was selected to balance mechanical integrity (larger mass yielding a stiffer holder) with tear resistance (smaller mass yielding a thinner film that can tear more easily). After curing at room temperature (22–25 °C) for ≥2 days, the PDMS sheet was demolded and punched to obtain the circular PDMS holder (e.g., 15 mm outer diameter); the sensing cavity itself (10 mm diameter; 0.6 mm depth) was defined by the mold protrusion (Figure S2A).

Experimental Section S3. Gas-mixing setup and H_2(g) concentration measurement. Gas-mixing procedures followed the gas-flow configuration described in our prior work.[S1] Calibration data relating rotameter scale readings to volumetric flow rates for N_2(g) and H_2(g) were provided by the manufacturer (Omega Engineering; Table S1 and Figure S3). A schematic of the gas-flow apparatus used for H_2(g) calibration and balloon experiments is shown in Figure S1. The manifold incorporated a PTFE 3-way valve that directed the mixed gas stream either (i) to a vent line for amperometric verification of H_2 levels and/or balloon filling, or (ii) to the experimental line used to supply gas to the test fixture. Amperometric measurements were obtained using a Unisense H_2 microsensor (H2-ST-1/4-304204, Unisense) with a Unisense Microsensor Multimeter, and the relationship between the sensor potential (mV) and the dissolved H_2(g) concentration was established using the calibration shown in Figure S4 (see also Table S8).[S2] In this work, 'H_2-equivalent dissolved concentration' ($[H_2]_{eq}$, μM) is defined as the apparent aqueous H_2 concentration obtained by converting the microsensor potential using the aqueous calibration (Figure S4) curve. This notation is used throughout, including for measurements performed under gas-phase exposure conditions. For the H_2(g)-filled balloon and chicken-skin configurations, H_2(g) measurements were acquired using the same amperometric sensor (Figures S5 and S6, respectively). The sensor settings were as follows: pre-amplifier range (0.1 mV pA^{-1}), polarization (100 mV), offset (0 mV), and gain (1.0).

Experimental Section S4. Stability study design and statistical analysis. Assembled sensors were stored at room temperature (22 °C), in a refrigerator (2.4 °C), or in a freezer (−17.3 °C) and evaluated after 1, 2, 3, and 4 weeks. For each storage temperature and time point, three independent samples ($n = 3$) were prepared and analyzed. The response from each stored sample was normalized to the corresponding fresh-sensor response, which was defined as 1.0. Statistical analysis was performed in OriginPro (Version 2026). For each temperature condition, differences across storage time were evaluated using one-way ANOVA followed by Tukey's multiple-comparisons test. A significance threshold of $\alpha = 0.05$ was used. Data are reported as mean ± standard deviation (SD).

Experimental Section S5. Stability of the sensing mixture (H_2(g) reactivity) under different storage conditions. To assess preservation of reactivity of the visual H_2(g) sensing mixture during storage, PDMS holders containing the sensing mixture were stored at room temperature (22 °C), in a refrigerator (2.4 °C), or in a freezer (−17.3 °C) for 1–4 weeks. Each holder was covered in a 60 mm Petri dish during storage. For each temperature–time condition, three samples were stored to allow assessment of reproducibility. After storage, the reactivity of the visual H_2(g) sensing mixture was evaluated using the balloon-based H_2(g) exposure experiment and RGB analysis described below.

In a separate test, two PDMS holders containing freshly prepared $PdCl_2$/PEG-40 HCO sensing mixture were placed in two covered 60-mm Petri dishes, and each covered Petri dish was then placed individually into a Mylar bag, vacuum-sealed by a Wallaby Impulse sealer, and stored at room temperature for approximately 60 days. Images of the sensing mixtures were acquired before and after storage, and analyzed by the same RGB analysis procedure described in the main text. Normalized brightness was calculated as the average RGB value of the sensing mixture divided by the average RGB value of the local background. Values are reported as mean ± SD from two independent samples.

Experimental Section S6. Balloon-based test of H_2(g) reactivity. A hydrogen-filled balloon was used as a controlled, convenient H_2(g) source for evaluating H_2(g) sensing film reactivity, rather than conducting animal tests, which are more complex, costly, and time-limited, as in a prior mouse study using a Mg implant.[S3] As H_2(g) permeates through the balloon latex very slowly, the H_2(g)-filled balloon serves as a simple, reproducible H_2(g) source. Each balloon was filled with ~1.7 L of H_2(g), sealed, and then used as the H_2(g) exposure source during imaging. The H_2 level at the balloon surface was verified at multiple locations and contact depths using the amperometric H_2 microsensor (H2-ST-1/4-304204, Unisense). With 100% H_2(g) inside the balloon, the amperometric sensor typically measured ~9–11 mV, corresponding to the H_2-equivalent dissolved concentration of ~8 µM. The visual sensing film (or sensing mixture in a PDMS holder) was placed on the balloon surface and the resulting color change was recorded by a smartphone camera. Apparent reaction kinetics were quantified from the time-dependent optical response using the RGB-based analysis described below. White balloons were used to facilitate background normalization for brightness-based image analysis.

Experimental Section S7. Detection of H_2(g) from Mg alloy degradation through chicken-skin barrier. A $MgGd_5$ alloy disk (5 mm diameter, 2 mm thickness) was fixed to the bottom of a Corning Falcon bacteriological Petri dish (CLS351008; 35 mm × 10 mm) using double-sided, double-lined silicone adhesive tape. The specimen was immersed in 4.0 mL of 1× PBS. Chicken leg skin was used as a diffusion barrier placed over the $MgGd_5$ alloy surface (not submerged in PBS) to separate the aqueous corrosion source from the air-facing sensing interface and thereby introduce transport-limited H_2(g) detection conditions. Fresh chicken leg skin was obtained from a local grocery store and used on the day of purchase; no mechanical thinning or chemical treatment was performed before use. Prior to use, the skin was gently rinsed with deionized water, blotted dry, and cut to fully cover the Petri-dish opening. The skin was

wrapped over the dish opening and positioned to cover the $MgGd_5$ alloy surface without submersion in PBS. The PBS volume (4.0 mL) was selected to avoid overflow such that additional perimeter sealing was not required. Chicken skin and other animal-skin membranes are commonly used as practical surrogate barriers in skin-interfacing/permeation studies when human tissue is not available.[S4-S6] In this work, chicken skin was used as a convenient biologically derived diffusion barrier to introduce transport-limited H_2(g) detection conditions, rather than to quantitatively replicate human transdermal flux. The bandage-type visual H_2(g) sensing film was attached to the air-exposed outer surface of the chicken skin above the PBS-immersed $MgGd_5$ specimen. Local H_2(g) levels on the chicken-skin were monitored using the amperometric H_2(g) microsensor and converted to H_2(g)-equivalent dissolved concentrations ($[H_2]_{eq}$, µM) using the calibration curve (Figure S4). Representative potential traces and the resulting concentration range for the chicken-skin setup are shown in Figure S6 (typically 145.4–186.1 µM under the conditions used here). The color change of the H_2(g) sensing film was recorded using a smartphone camera under fixed position and constant ambient lighting. For quantitative analysis, a consistent region of interest (ROI) was applied across time points and samples, and RGB/brightness values were extracted using the method described below. The normalized brightness ratio (B_t/B_0) was calculated, where B_0 is the initial brightness at t = 0 and B_t is the brightness at time t, and the brightness change was expressed as $\Delta B = 1 - (B_t/B_0)$. Apparent kinetics were compared using the time required for B_t/B_0 to decrease from 1.0 to 0.6; linear regression over this fixed window was used to obtain an empirical rate for each site of measurement. $MgGd_5$ was selected because Mg–rare earth alloys, including MgGd-based systems, have been investigated for biodegradable implant applications. [S7]

Experimental Section S8. Preparation of balloons with various %H_2(g) for testing. Controlled H_2(g)/N_2(g) mixtures (11.7%, 24.6%, 47.6%, and 100% H_2(g) by volume) were prepared by adjusting the individual H_2 and N_2 rotameter scale settings on the gas-delivery system. Scale readings were converted to volumetric flow rates (mL min^{-1}) using the manufacturer-provided calibration curve (Figure S3). Each balloon was filled to the same total gas volume by controlling the total flow rate and fill time (Table S6). Maintaining a constant volume minimized differences in balloon pressure and gas-delivery conditions between compositions, enabling fair comparison across the %H_2(g) range.

Experimental Section S9. Balloon-surface optical measurement and image analysis for LOD determination. Immediately after filling with H_2(g), the sealed balloon was used as the substrate for measurements. The H_2(g) sensing film was placed directly on the balloon surface under constant lighting and fixed position. Time-lapse images were acquired. RGB-based image analysis was used to quantify the optical response based on the normalized brightness ratio (B_t/B_0). ΔB values at 10, 20, and 30 min after placing the film on the balloon surface were used for LOD determination. The corresponding response curves and LOD summaries are provided in Figure S8 and Table S5. For each balloon composition, three independent measurements were performed unless otherwise noted.

Experimental Section S10. Effect of H_2(g) flow rate on the H_2(g) sensing film's optical response kinetics. To evaluate whether the optical response of the H_2(g) sensing film depends on gas flow rate, 100% H_2(g) was delivered to a H_2(g) sensing film placed at the port of a Franz-cell-based setup (Figure S1). Flow rate was controlled using a single-flow-tube rotameter set to predetermined positions corresponding to 10, 32, 61, and 89 mL min^{-1} (Table S7; manufacturer calibration provided by Omega Engineering). The flattened Franz cell was secured and covered with 3M 9834 medical tape to provide a sealed, flat mounting interface, on top of which the sensing film assembly was affixed. The sensing film was prepared by filling the sensing mixture into a PDMS holder such that the sensing mixture directly contacted the medical-tape surface. After initiating H_2(g) flow at the specified rate, images of the sensing area were acquired at defined time intervals (0–15 min). Image processing, brightness calculation, and normalization to B_t/B_0 were

performed as described previously. Three independent trials were performed for each flow-rate condition, and the value of mean ± standard deviation is reported. Analysis of covariance (ANCOVA) was used to test whether B_t/B_0 changed over time, whether the overall response differed across flow-rate conditions, and whether the time-dependent response pattern differed among flow rates. For ANCOVA, effects with $p < 0.05$ were considered statistically significant ($\alpha = 0.05$, 95% confidence level).

Figure S1. Gas-flow setup. The manifold mixes H_2 and N_2 using calibrated rotameters and directs the mixed stream via a 3-way valve either to a vent line for amperometric measurements and balloon filling or to the experimental line supplying the test fixture.

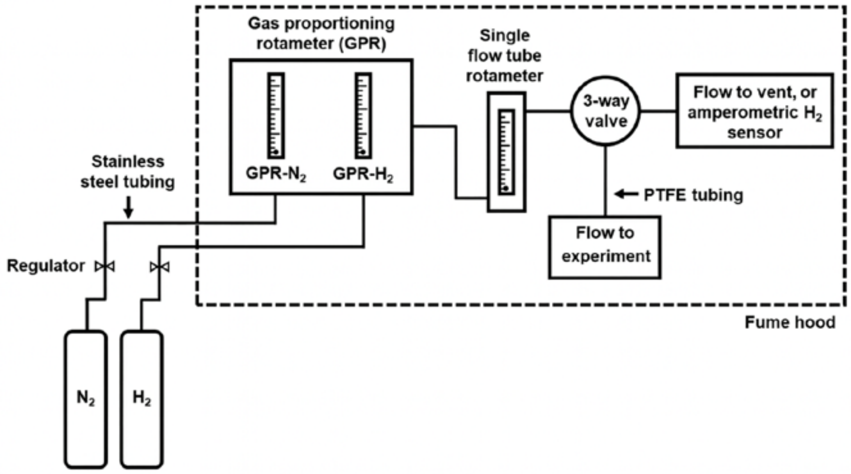

Table S1. Manufacturer-provided rotameter calibration for converting scale readings to volumetric flow rates of H_2 and N_2 used to prepare defined gas mixtures. Theoretical H_2-equivalent dissolved concentration ($[H_2]_{eq}$ (µM)), calculated from gas-phase flow ratios, are listed for reference.

**Hydrogen x value (scale reading)**	**Hydrogen flow rate (mL min⁻¹)**	**Nitrogen x value (scale reading)**	**Nitrogen flow rate (mL min⁻¹)**	**Theoretical Hydrogen concentration (µM)**
129	862	110	287	600
89	480	110	287	500
73	358.1	129	359	399
50	214.6	129	359	299.1
30	120	129	359	200.2
10	53.3	132	371	100.4

Figure S2. Aluminum plate mold and fabrication procedure for the PDMS thin-layer holder. (A) Schematic of the mold geometry defining the base diameter (d_{base} = 33 mm), protrusion diameter ($d_{protrusion}$ = 10 mm), and protrusion height ($h_{protrusion}$ = 0.6 mm), which set the diameter and depth of the sensing cavity in the cast PDMS holder. (B) Representative photographs of the molding steps: (1) aluminum mold, (2) mold positioned in a 6-well plate for alignment, (3) PDMS cast, degassed, and thermally cured on the mold, and (4) demolded PDMS sheet followed by punching to obtain the circular holder.

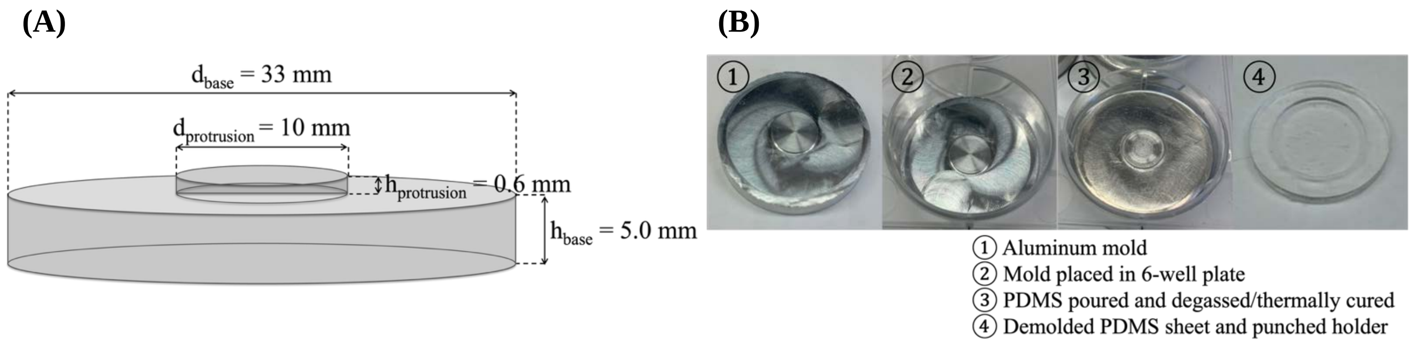

Figure S3. Manufacturer-provided rotameter calibration curves for the H_2 and N_2 flow tubes used to convert rotameter scale readings to volumetric flow rates for preparing defined gas mixtures (see Table S1). Second-order polynomial fits were used to convert scale reading (x, mm) to flow rate (y, mL min^{-1}): H_2, $y = 0.035x^2 + 1.93318x + 30.48352$ (R^2 = 0.99977); N_2, $y = 0.00994x^2 + 1.41447x + 11.51912$ (R^2 = 0.99955).

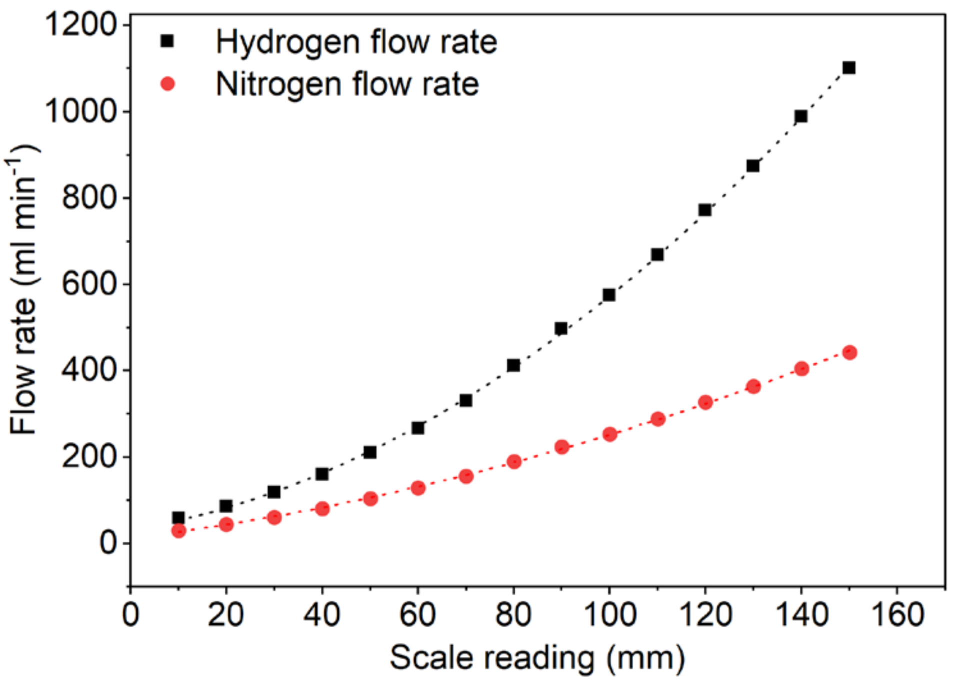

Figure S4. Amperometric H_2 microsensor calibration used to convert measured potential (mV) to H_2-equivalent dissolved concentration ($[H_2]_{eq}$, µM) over 0–600 µM. (A) Calibration data (mean ± SD) collected across 11 measurements over ~3 weeks; the concentration conversion was performed using a second-order polynomial fit, Potential (mV) = $0.00094[H_2]^2 + 2.2[H_2] - 7.5$ ($R^2 = 0.9997$). (B) Residual analysis comparing linear and second-order polynomial models; the polynomial fit reduced systematic deviations and yielded a lower root-mean-square error (RMSE) and was used for concentration conversion within the calibrated range (no extrapolation).

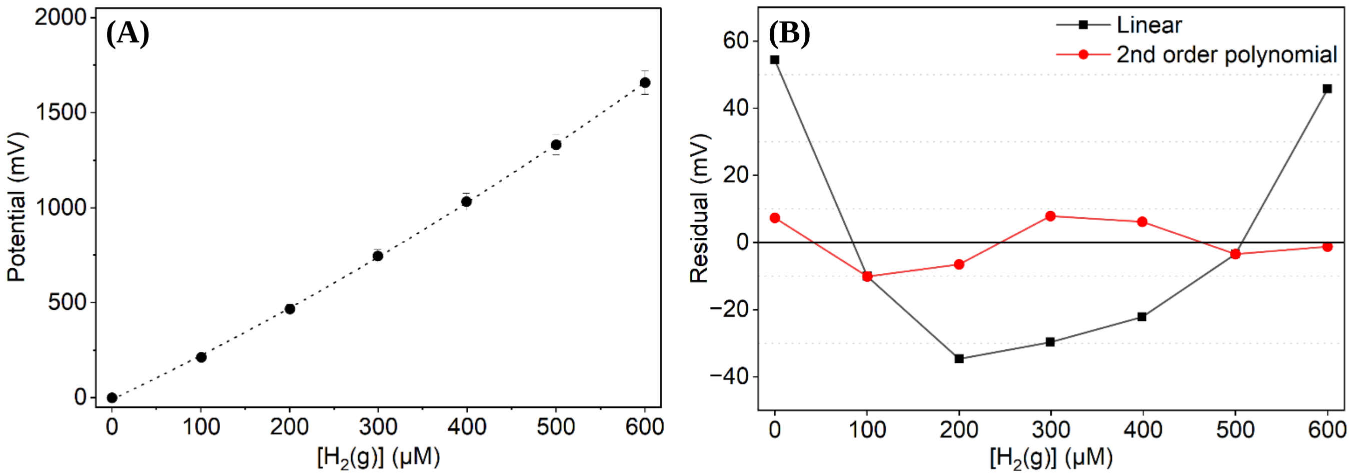

Figure S5. Representative potential traces measured on the surface of an H_2(g)-filled balloon at multiple contact locations/depths using the amperometric H_2 microsensor. The time axis started after signal stabilization. ($[H_2(g)]$ = 7.59 to 8.84 µM). The corresponding H_2-equivalent dissolved concentration ($[H_2]_{eq}$, µM) was obtained using the aqueous calibration shown in Figure S4.

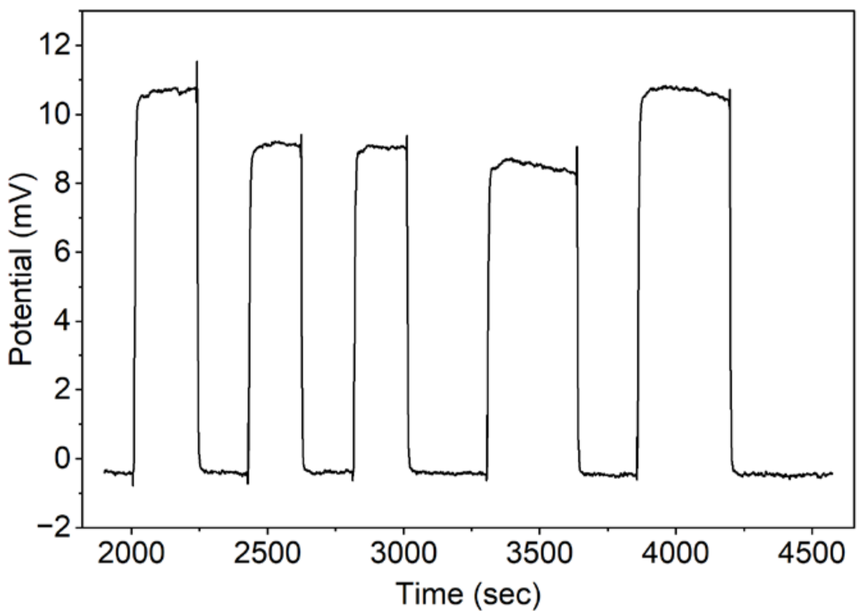

Figure S6. Potential measured on the outer surface of chicken leg skin covering a $MgGd_5$ specimen immersed in 1× PBS, acquired using the amperometric H_2 microsensor. The corresponding H_2-equivalent dissolved concentration ($[H_2(g)]_{eq}$, μM) was obtained using the calibration in Figure S4. ($[H_2(g)]_{eq}$ = 145.4 to 186.1 μM)

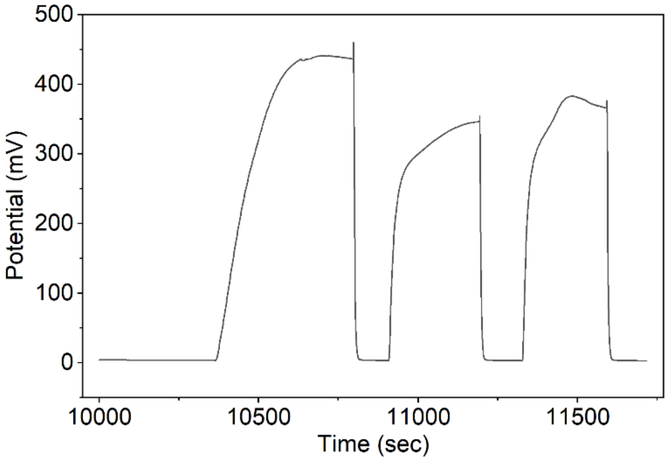

Table S2. Analysis of the normalized optical response of the visual $H_2(g)$ sensing film. Linear regressions of B_t/B_0 versus time were performed over two consecutive time intervals (0–3 and 3–6 min) to extract apparent response rates (slopes) and goodness-of-fit (R^2). This segmentation enables direct comparison of early-stage kinetics and assessment of rate acceleration consistent with an auto-catalytic $PdCl_2$ reduction pathway.

Reaction time (min)	0 - 3	3 - 6
Linear equation	y=-0.00071x + 0.999	y=-0.0013x + 1.054
R^2	0.99	0.98

Table S3. Storage-temperature-dependent stability of the normalized optical response (Fresh = 1.000). Values are reported as mean ± SD (n = 3). Statistical comparisons within each temperature condition were performed using one-way ANOVA followed by Tukey's multiple-comparisons test (overall ANOVA p-values: room temperature, p = 0.00197; refrigerator, p = 0.02203; freezer, p = 0.33302). P-values shown in the table are Tukey-adjusted comparisons versus Fresh; asterisks (*) indicate statistically significant differences from Fresh ($p < 0.05$).

**Storage temperature**	**Fresh**	**Week 1**	**Week 2**	**Week 3**	**Week 4**
**Room temperature (22 °C)**	1.000	0.9667 ± 0.0208 (p = 0.72482)	0.9233 ± 0.0551 (p = 0.09498)	0.8533 ± 0.0379 (p = 0.00189*)	0.8933 ± 0.0208 (p = 0.01671*)
**Refrigerator (2.4 °C)**	1.000	0.9700 ± 0.0100 (p = 0.59382)	0.9867 ± 0.0252 (p = 0.96084)	0.9400 ± 0.0265 (p = 0.08248)	0.9267 ± 0.0404 (p = 0.02970*)
**Freezer (−17.3 °C)**	1.000	0.9533 ± 0.0577 (p = 0.64739)	0.9900 ± 0.0529 (p = 0.99799)	0.9400 ± 0.0361 (p = 0.43096)	0.9467 ± 0.0322 (p = 0.53584)

Table S4. H_2(g) reactivity of the visual H_2(g) sensing films after storage, quantified by linear fitting of the normalized brightness response. Experiments were performed in triplicate (n = 3). For each condition, B_t/B_0 was fitted over the initial 0–6 min window to obtain a linear equation, where the slope represents the apparent response rate. The Fresh sample fit (y= -0.086x + 0.93, R^2 = 0.97) is provided as the baseline reference for comparing rate changes of the H_2(g) sensing films after 1–4 weeks of storage at respective temperatures.

		Storage time			
		1 week	2 weeks	3 weeks	4 weeks
Temperature (°C)	22	y= -0.064x + 1.002	y= -0.058x + 0.98	y= -0.048x + 0.97	y= -0.061x + 0.95
		**R^2** = 0.99	**R^2** = 0.98	**R^2** = 0.98	**R^2** = 0.96
	2.4	y= -0.069x + 1.03	y= -0.076x + 1.02	y= -0.071x + 1.04	y= -0.073x + 1.02
		**R^2** = 0.99	**R^2** = 0.99	**R^2** = 0.99	**R^2** = 0.99
	-17.3	y= -0.080x + 0.95	y= -0.08x + 1.00	y= -0.084x + 0.97	y= -0.074x + 0.99
		**R^2** = 0.96	**R^2** = 0.98	**R^2** = 0.96	**R^2** = 0.98

Figure S7. Test of H_2-sensing mixtures stored in vacuum-sealed Mylar bags at room temperature. Representative images of two sensing-mixture samples stored in covered 60-mm Petri dishes stored in vacuum-sealed Mylar bags before and after approximately 60 days at room-temperature are shown. Normalized brightness was calculated as the average RGB value of the sensing mixture divided by the average RGB value of the local background and reported as mean ± SD from two independent samples.

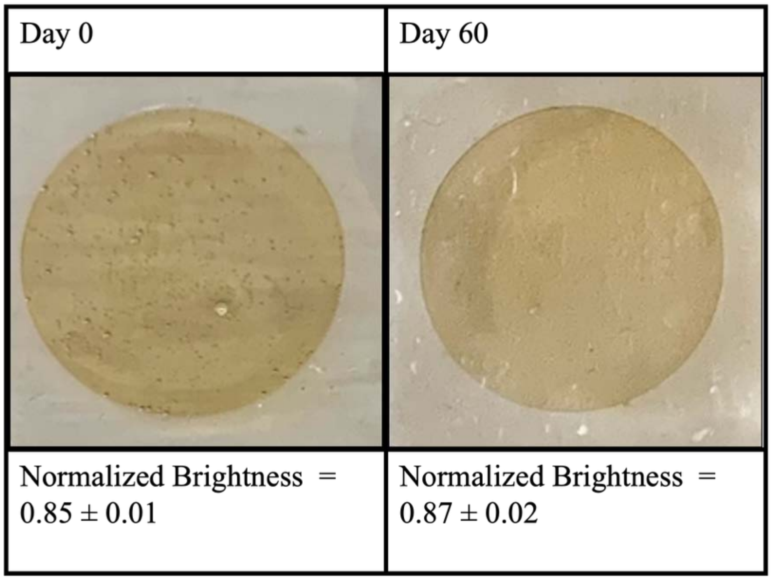

Figure S8. Time-dependent normalized brightness response (B_t/B_0) of the visual H_2(g) sensing film under 0–100% H_2(g) used for LOD analysis. Data are shown as mean ± SD (n = 3). Brightness changes were evaluated over different response windows (e.g., 0–10, 0–20, and 0–30 min) and concentration ranges; resulting linearity metrics and LOD values are summarized in Table S5.

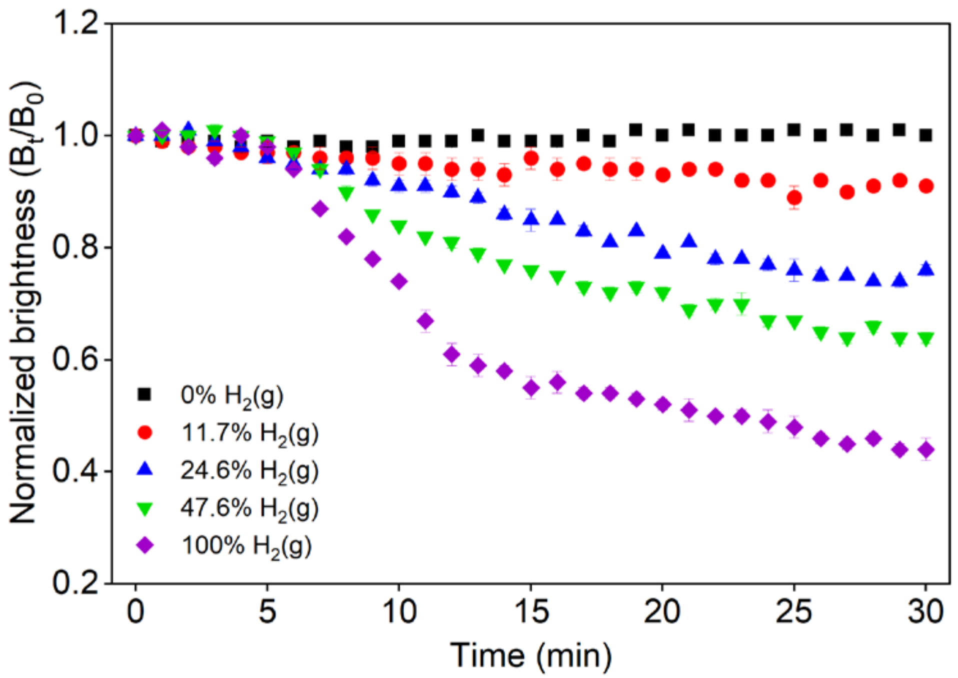

Table S5. Linearity metrics (RMSE, R^2) and IUPAC limit of detection (LOD) calculated from ΔB for different response windows (0–10, 0–20, and 0–30 min) and calibration ranges (0–24.6, 0–47.6, and 0–100% H_2(g)). Experiments were performed in triplicate (n = 3). LOD was computed as LOD = 3σ_blank/m, where σ_blank is the standard deviation of ΔB at 0% H_2(g) within each response window, and m is the slope of the corresponding linear calibration curve.

Time window (minutes)	Range (%)	σ_blank	Slope (m)	Intercept (b)	RMSE (ΔB)	R^2	LOD (IUPAC) (%H_2(g))
0–10	0–24.6	0.0037	0.0035	0.0066	0.0011	0.9991	3.20
0–10	0–47.6	0.0037	0.0032	0.0088	0.0025	0.9981	3.43
0–10	0–100	0.0037	0.0025	0.0209	0.0128	0.9800	4.39
0–20	0–24.6	0.0028	0.0085	-0.0106	0.0143	0.9725	0.98
0–20	0–47.6	0.0028	0.0061	0.0117	0.0270	0.9401	1.38
0–20	0–100	0.0028	0.0046	0.0359	0.0349	0.9566	1.80
0–30	0–24.6	0.0017	0.0101	-0.0124	0.0101	0.9903	0.49
0–30	0–47.6	0.0017	0.0079	0.0083	0.0240	0.9712	0.63
0–30	0–100	0.0017	0.0055	0.0494	0.0477	0.9427	0.90

Table S6. Gas-flow settings used to prepare different $H_2(g)/N_2(g)$ mixtures for balloon-based experiments. H_2 and N_2 flow rates were set using calibrated rotameters (Table S1; Figure S3) and applied for balloon filling under identical conditions (balloon size and filling procedure held constant) to ensure consistent headspace composition across trials.

**Target $H_2(g)$ (vol%)**	**Flow meter scale setting: $H_2(g)$**	**Flow meter scale setting: $N_2(g)$**	**Calculated flow rate of $H_2(g)$ (mL min^{-1})**	**Calculated flow rate of $N_2(g)$ (mL min^{-1})**	**Total flow rate (mL min^{-1})**	**Fill time (min)**	**Total gas volume in balloon (mL)**
11.7	10	150	58.5	443	501.5	3.5	1755.25
24.6	30	130	119	364	483	3.6	1738.8
47.6	70	130	330	364	694	2.5	1735
100	100	0	576	0	576	3	1728

Figure S9. Time-course of the normalized brightness response (B_t/B_0) of the $H_2(g)$ sensing film under 100% $H_2(g)$ at four flow rates (10, 32, 61, and 89 mL min^{-1}) over 0–15 min. Data are shown as mean ± SD (n = 3). Analysis of covariance (ANCOVA) indicated a significant effect of time ($p < 0.0001$), but no significant effect of flow rate ($p = 0.218$) or time × flow-rate interaction ($p = 0.246$), suggesting that the optical responses were statistically indistinguishable across the tested flow rates.

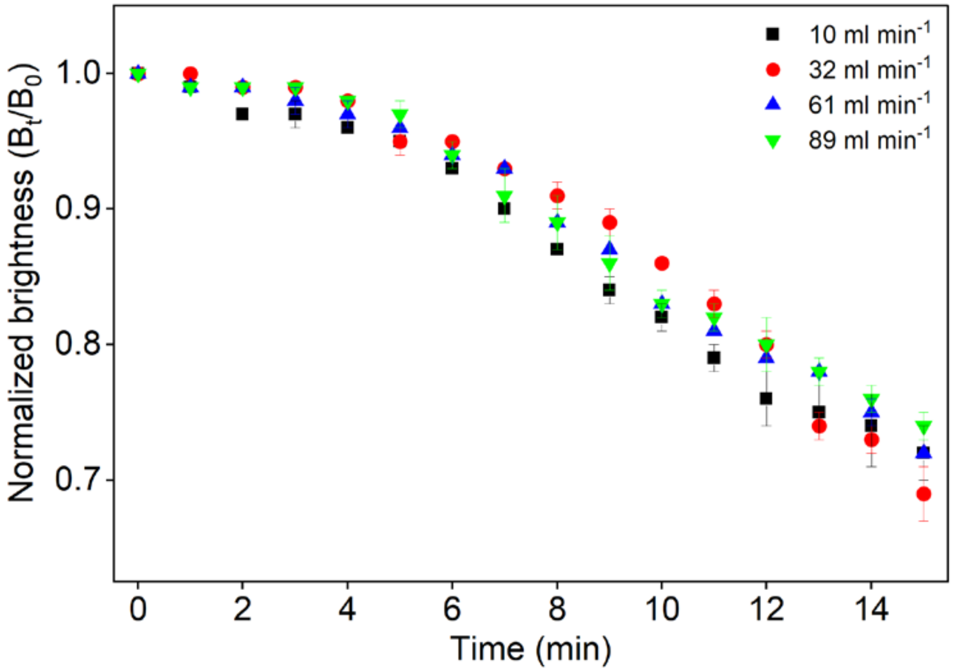

Table S7. Conversion of single-flow-tube rotameter scale readings to 100% H_2(g) volumetric flow rates used in the flow-rate dependence experiment. Scale-reading–to–flow-rate values were provided by Omega Engineering for the rotameter in the gas-delivery line shown in Figure S1.

**Single flow tube rotameter scale reading (mm)**	**H_2(g) flow rate (mL min^{-1})**
0.5	10
3.0	32
6.0	61
8.0	89

Table S8. Balloon-based calibration linking %H_2(g) to H_2-equivalent dissolved concentration, ($[H_2]_{eq}$, µM), obtained using the amperometric H_2 microsensor calibration (Figure S4). Known H_2(g) compositions (11.7, 24.6, 47.6, and 100%) were prepared in identical 10-inch balloons, and potentials at balloon surface were recorded using the amperometric H_2 microsensor (mean ± SD, n = 3). [H_2] values were obtained from the measured potential using the calibration in Figure S4.

**% H_2(g)**	**Potential (mV), mean**	**Potential (mV), SD**	**[H_2] (µM), mean**	**[H_2] (µM), SD**
11.7	4.43	0.86	5.41	0.4
24.6	5.37	1.66	5.83	0.74
47.6	12.17	1.72	8.91	0.78
100.0	23.0	3.84	13.78	1.72

Table S9. H_2-equivalent concentration ($[H_2]_{eq}$ (µM)) measured under different 100% H_2(g) flow rates in the Franz-cell test fixture as a reference dataset illustrating the dependence of measured signal on gas flow conditions. Potentials were measured with the amperometric H_2 microsensor and converted to $[H_2]$ (µM) using the calibration in Figure S4 (mean ± SD, n = 3).

**Flow rate (mL min^{-1})**	**Potential (mV), mean**	**Potential (mV), SD**	**$[H_2]$ (µM), mean**	**$[H_2]$ (µM), SD**
10	352.5	7.78	153.56	3.12
32	940.0	4.24	371.66	1.46
61	1340.5	7.78	504.13	2.48
89	1521.0	7.08	560.53	2.18

REFERENCES

(S1) Smith, M. E.; Rose, D. P.; Cui, X.; Stastny, A. L.; Zhang, P.; Heineman, W. R. A Visual Hydrogen Sensor Prototype for Monitoring Magnesium Implant Biodegradation. *Anal. Chem.* **2021**, *93*, 10487–10494.

(S2) Kuhlmann, J.; Witte, F.; Heineman, W. R. Electrochemical Sensing of Dissolved Hydrogen in Aqueous Solutions as a Tool to Monitor Magnesium Alloy Corrosion. *Electroanalysis.* **2013**, *25*, 1105–1110.

(S3) Noviana, D.; Paramitha, D.; Ulum, M. F.; Hermawan, H. The Effect of Hydrogen Gas Evolution of a Magnesium Implant on Postimplantation Mortality of Rats. *J. Orthop. Transl*. **2016**, *5*, 9–15.

(S4) Lachner, J.; Derdak, S.; Mlitz, V.; Wagner, T.; Holthaus, K. B.; Ehrlich, F.; Mildner, M.; Tschachler, E.; Eckhart, L. An In Vitro Model of Avian Skin Reveals Evolutionarily Conserved Transcriptional Regulation of Epidermal Barrier Formation. *J. Invest. Dermatol.* **2021**, *141*, 2829–2837.

(S5) Neupane, R.; Boddu, S. H. S.; Renukuntla, J.; Babu, R. J.; Tiwari, A. K. Alternatives to Biological Skin in Permeation Studies: Current Trends and Possibilities. *Pharmaceutics.* **2020**, *12*, 152.

(S6) Abd, E.; Yousef, S. A.; Pastore, M. N.; Telaprolu, K.; Mohammed, Y. H.; Namjoshi, S.; Grice, J. E.; Roberts, M. S. Skin models for the testing of transdermal drugs. *Clin. Pharmacol. Adv. Appl.* **2016**, *8*, 163–176.

(S7) Niranjan, C. A.; Raghavendra, T.; Rao, M. A.; Siddaraju, C.; Gupta, M.; Jain, V. K. S.; Aishwarya, R. Magnesium Alloys as Extremely Promising Alternatives for Temporary Orthopedic Implants—A Review. *J. Magnes. Alloys.* **2023**, *11*, 2688–2718.
